# Chromium (VI) Uptake and Tolerance Potential in Cotton Cultivars: Effect on Their Root Physiology, Ultramorphology, and Oxidative Metabolism

**DOI:** 10.1155/2014/975946

**Published:** 2014-05-14

**Authors:** M. K. Daud, Lei Mei, M. T. Variath, Shafaqat Ali, Cheng Li, M. T. Rafiq, S. J. Zhu

**Affiliations:** ^1^Department of Agronomy, College of Agriculture and Biotechnology, Zhejiang University, Zijingang Campus, Hangzhou 310058, China; ^2^Department of Biotechnology and Genetic Engineering, Kohat University of Science and Technology, Kohat 26000, Pakistan; ^3^International Crops Research Institute for the Semi-Arid Tropics (ICRISAT), Hyderabad 502324, India; ^4^Department of Environmental Sciences, Government College University, Faisalabad 38000, Pakistan; ^5^Ministry of Education Key Laboratory of Environmental Remediation and Ecological Health, College of Environmental and Resource Sciences, Zhejiang University, Hangzhou 310058, China

## Abstract

Chromium (Cr) is present in our environment as a toxic pollutant, which needs to be removed using phytoremediation technology. In present study, two transgenic cotton cultivars (J208, Z905) and their hybrid line (ZD14) were used to explore their Cr uptake and tolerance potential using multiple biomarkers approach. Four different levels of Cr (CK, 10, 50, and 100 **μ**M) were applied. Cr caused a significant reduction in root/shoot length, number of secondary roots, and root fresh and dry biomasses at 100 **μ**M. Cr accumulated more in roots and was found higher in hybrid line (ZD14) as compared with its parent lines (J208, Z905) at all Cr stress levels (10, 50, and 100 **μ**M). Cr translocation was less than 1 in all cultivars. Ultrastructural studies at 100 **μ**M Cr showed an increase in number of nuclei and vacuoles and presence of Cr dense granules in dead parts of the cell (vacuoles/cell wall). Malondialdehyde (MDA), hydrogen peroxide (H_2_O_2_), total soluble proteins, superoxide dismutase (SOD), peroxidase (POD), ascorbate peroxidase (APX), catalase (CAT), and glutathione reductase (GR) as a whole were upregulated with elevated levels of Cr. Higher Cr uptake by roots, accelerated metabolism, and Cr sequestration in dead parts of the cell indicate that these cotton cultivars can be useful for Cr accumulation and tolerance.

## 1. Introduction

Chromium (Cr) like other heavy metals threatens both crop production and human health. To minimize its concentration in environment, various reclamation technologies such as redox processes, ion exchange, and reverse osmosis [1] have been introduced. However, most of them are highly expensive and cumbersome. They need to be replaced by cheaper and more cost-effective plant-based technologies. They are, for example, phytoextraction, phytostabilization, and phytoremediation.

Cr-mediated ground water and soil pollution need to be controlled either by avoiding its spills from various industries such as plating, alloying, tanning of animal hides, inhibition of water corrosion, and textile dyes [2] or by exploiting plant-based phytoextraction technologies. Unlike other toxic pollutants, Cr has several oxidation states. Among these, the trivalent Cr (III) and the hexavalent Cr (VI) are the most abundant. Since both possess different chemical, toxicological, and epidemiological feature, they are differently regulated by Environmental Protection Agency (EPA) [3]. Cr (III) is an essential trace element for human and animal health [4], being necessary for sugar and lipid metabolism [5]. It occurs in the form of oxides, hydroxides, and sulfates [6, 7]. It has relatively less mobility in soil and water due to its binding tendency towards organic matters. It is rapidly oxidized to Cr (VI) in acidic soils in the presence of Mn, which becomes insoluble in water and alternatively has adverse effects on plant growth and development [8]. Cr (VI) is more toxic than Cr (III) for human beings, plants, aquatic animals, and microorganisms [9]. Due to these properties, Cr (VI) is a priority pollutant [6, 10] among the scientific community.

Cr mostly resides in roots and only a fraction of it is translocated to the shoots [11, 12]. Concentration of Cr in roots is 100-fold higher than the shoots [13] in most cases. Various factors such as pH, oxidative state of Cr and its concentration, salinity, and the presence of dissolved salts play an active role in its uptake from hydroponic media [14]. Upon entry into plants, Cr uses channels of sulfur and iron for upward translocation, which results in silent competition among the three. Resultantly, Cr is mainly confined to roots. This competition seems independent of the form of Cr tested [9].

In plants, Cr induces numerous physiological, biochemical, and ultrastructural alterations. Growth and biomass reduction, chlorosis in young leaves, lowering of pigment contents, disturbance of stomatal conductance alteration of enzymatic function, and damage to root cells and ultramorphological modifications [15, 16] in roots and leaves are some of the reported adverse effects of Cr in plants. Roots are severely damaged due to their direct contact with Cr. For example, shortening and browning of roots as well as presence of less number of root hairs in* Zea mays *L. [17] and adverse effects on lateral roots development and root number in mung bean [18] have been reported.

Very few reports cite strong cytotoxic nature of Cr (VI) in plants. Fasulo et al. [19] observed inhibition of cell proliferation and the presence of multinucleated, highly vacuolated giant cells in* Euglena gracilis*. Mouvet [20] has reported electron-dense bodies in cell walls, cytoplasm, and vacuoles in* Fontinalis antipyretica *L., grown in river water contaminated with Cr and Cu. Condensed and irregular structural features of root tips in bush bean as well as damage to membranes especially the tonoplast have been observed by [21]. Panda [22] found plasmolysed cell, large vacuoles, and presence of dense lysosomes like organelles in Cr (VI) treated roots.

All such types of anomalies result in the production of reactive oxygen species (ROS) [23, 24]. ROS can cause oxidative damage to cell membranes, chlorophyll pigments, lipids, nucleic acids, and proteins [8], which result in altered metabolism and finally cell death. However, plants are capable of mediating the deleterious effects of these ROS [22] by activating an extensive network of antioxidants. It is comprised of various enzymes, such as superoxide dismutase (SOD), peroxidase (POD), ascorbate peroxidase (APX), and low molecular weight antioxidants, such as glutathione [25]. Imbalance between rate of ROS production and antioxidant system activity causes ROS accumulation in plants [8]. Cr-mediated ROS production can be either by direct electron transfer or by inhibition of metabolic reactions [26].

Cotton has been almost unexploited for heavy metal tolerance and accumulation. It can be a potential candidate for heavy metal such as Cr pollution control. Due to rapid cultivation of transgenic cotton cultivars in the industrialized area of the world, it is foremost needed to exploit them for Cr remediation in Cr-polluted areas.

We planned a comprehensive study to compare the Cr uptake potential of two transgenic cotton cultivars (insect and herbicide resistant) and their hybrid line testing various biomarkers as their “quantitative responses” towards Cr stress levels. Biometric parameters such as length, biomass, and tolerance index were measured as indices of metabolic damage. Cr contents levels were determined in both roots and leaves so as to evaluate their accumulation potential. Ultramorphological studies were performed to locate possible Cr detoxification and accumulation sites. Oxidative stress biomarkers such as protein, MDA, and H_2_O_2_ were studied to know the extent of Cr-induced oxidative damage in roots. And moreover, their antioxidative metabolism was evaluated to explore their possible utilization in remediation of Cr.

## 2. Materials and Methods

### 2.1. Plant Materials and Culturing Methods

In our present experiment, we used two transgenic cotton cultivars (J208, Z905) and their hybrid line (ZD14). J208 is an herbicide resistant transgenic cotton cultivar and Z905 is an insect resistant transgenic cotton cultivar. Lint was removed with concentrated hydrogen sulfide (H_2_SO_4_) followed by constant washing with tap water for about 30 min. Uniform-sized seeds with shining color were first immersed in 70% ethanol followed by washing 3 times with double distilled water (ddH_2_O). Then seeds were surface-sterilized with 0.1% HgCl_2_ solution for 8–10 min, which were washed for 3-4 times first with distilled water (dH_2_O) and ddH_2_O. Seeds were soaked for 4 hr in dH_2_O at temperature 35°C. After that they were sown in presterilized sand in growth chamber for seven days. Temperature of the growth chamber was 28 ± 2°C, relative humidity was kept 60%, and light and dark photoperiod was 16:8 hr. Uniform-sized seven-day-old seedlings were transferred to green house by maintaining 32 ± 2°C day and 30 ± 2°C night temperature, while the light and dark period was 14:10 hr. Seedlings were grown for seven days in adaptation media. The nutrients media composition was 500 *μ*M (NH_4_)_2_SO_4_, 500 *μ*M MgSO_4_, 200 *μ*M K_2_SO_4_, 1000 *μ*M KNO_3_, 600 *μ*M Ca(NO_3_)_2_·4H_2_O, 200 *μ*M KH_2_PO_4_, 100 *μ*M Na_2_-EDTA, 10 *μ*M FeSO_4_·7H_2_O, 0.5 *μ*M MnSO_4_·H_2_O, 0.25 *μ*M ZnSO_4_·7H_2_O, 0.05 *μ*M CuSO_4_·5H_2_O, 100 *μ*M H_3_BO_3_, and 0.02 *μ*M (NH_4_)_6_Mo_7_O_24_.

### 2.2. Cr Treatment Process and Measurement of Growth Performance Parameter

14-day-old seedlings were subjected to exceeding levels of Cr (VI) in the form of potassium dichromate (K_2_Cr_2_O_7_). Four levels of Cr (*μ*M) were kept, that is, 0, 10, 50, and 100. Each level was randomly replicated thrice. Each replication consisted of 5 L pot having covered polystyrol plates with seven evenly spaced holes. In each hole, two seedlings were kept. Thus each replication consisted of 14 seedlings and there were 42 plants per treatment. Nutrients solution was constantly aerated with air pump and after every three days solution was changed with the new one. pH of the culture media was maintained between 5.6 and 5.7 either with 0.1 M HCl or NaOH and was adjusted every other day. Treatment regime was maintained for 7 days. At the end of the treatment, 21-day-old seedlings were subjected to the measurement the growth-related parameters such as roots, shoot lengths, root fresh and dry biomass, and biomass-based root growth inhibition. For length measurement, single randomly selected plant per replication was taken. While for the root biomass determination and root biomass-based inhibition, three randomly selected plants per replication were taken.

### 2.3. Determination of Cr Contents in Roots and Leaves

Cr contents were analyzed in both roots and leaves using atomic absorption spectrometery (PE-100, PerkinElmer). Number of plants per replication was three. At the end of the experiment, seedlings from both nonstressed and Cr-stressed population were washed with tap and distilled water thrice, respectively. Roots were immersed in 20 mM EDTA-Na_2_ for 15–20 min, which were subsequently washed with dH_2_O for three-four times. Seedlings were separated into roots and leaves, oven-dried at 80°C for 48 hr, and then ground into powder. 0.2 g of each root and shoot sample were digested with a mixture of 5 mL of HNO_3_ + 1 mL of HClO_4_. The resultant solutions were diluted to 25 mL using 2% HNO_3_ and then filtered. The root and leaves filtrates were used for Cr analysis.

### 2.4. Root Specimen Preparation for Ultramicroscopic Observations

Root ultrathin sections were prepared for ultramorphological studies according to Daud et al. [27]. Briefly, root tips (~2-3 mm) of randomly selected plants of both control and 100 *μ*M Cr-treated were fixed overnight in 2.5% glutaraldehyde (v/v) in 0.1 M PBS (sodium phosphate buffer, pH 7.4). They were vacuum-infiltrated for 15 min and washed three times with the same PBS. The samples were post-fixed in 1% osmium (VIII) oxide (O_s_O_4_) for 1 hr and were washed three times in 0.1 M PBS (pH 7.4) with a ten-minute interval between each washing. After that, they were dehydrated in a graded ethanol series (50, 60, 70, 80, 90, 95, and 100%) with 15–20 min interval and finally by absolute acetone for 20 min. The samples were then infiltrated and embedded in Spurr's resin overnight. After heating the specimens at 70°C for 9 hr, the ultrathin sections (80 nm) were prepared and mounted on copper grids for viewing in the transmission electron microscope (JEOL TEM-1230EX) at an accelerating voltage of 60.0 kV.

### 2.5. Measurements of Lipid Peroxidation, Hydrogen Peroxide, and Total Soluble Protein

0.8 g roots materials of all three cultivars were used for the determination of lipid peroxidation, hydrogen peroxide, and total soluble proteins. Lipid peroxidation was estimated in terms of malondialdehyde (MDA) contents and was determined as 2-thiobarbituric acid (TBA) reactive substances following the method of [28]. For determination of hydrogen peroxide (H_2_O_2_) content [29], 0.8 g roots were crushed with 8.0 mL of trichloroacetic acid (TCA) (0.1%, w/v) in ice cold conditions and the homogenate was centrifuged at 14,000 g for 20 min. 4 mL reaction mixture contained 1 mL supernatant, 1 mL PBS, and 2 mL potassium iodide (KI) and the absorbance was read at 390 nm. H_2_O_2_ content was determined using an extinction coefficient of 0.28 *μ*Mcm^−1^ and expressed as *μ*mol g^−1^ FW.

In order to determine the total soluble proteins, 0.8 g roots sample was homogenized in 8 mL of 50 mM potassium phosphate buffer (PBS) (pH7.8) under chilled conditions. The crude extract was centrifuged at 14,000 g for 15 min at 4°C and the supernatant was used for the determination of total soluble protein using the method of [30] and bovine serum albumin was used as a standard.

### 2.6. Samples Preparation for Metabolic Antioxidant Assays

In both Cr nontreated and treated root samples, metabolic antioxidant assays were performed according to established protocols. For each assay, 0.8 g fresh weight of roots was taken and the extraction buffer (PBS 7.8) volume was 8 mL. Superoxide dismutase (SOD) (EC1.15.1.1) activity was determined according to [31] following the inhibition of photochemical reduction due to nitro blue tetrazolium (NBT). Ascorbate peroxidase (APX) (EC 1.11.1.11) activity was measured using the protocol of [32]. Catalase (CAT) (EC1.11.1.6) activity was measured as determined by [33], while for the determination of peroxidase (POD) (EC1.11.1.7) activity, the method of [34] was used. Glutathione reductase (GR) (EC1.6.4.2) activity was assayed according to [35].

### 2.7. Statistical Analyses

The data obtained were subjected to one-way analysis of variance (ANOVA) using STATIX9. All the results were the means ± SD of three replications. Means were separated by least significant difference (LSD) test at 5% level of significance.

## 3. Results

### 3.1. Cr Stress Differentially Inhibited Root Morphology

**Figure 1:**
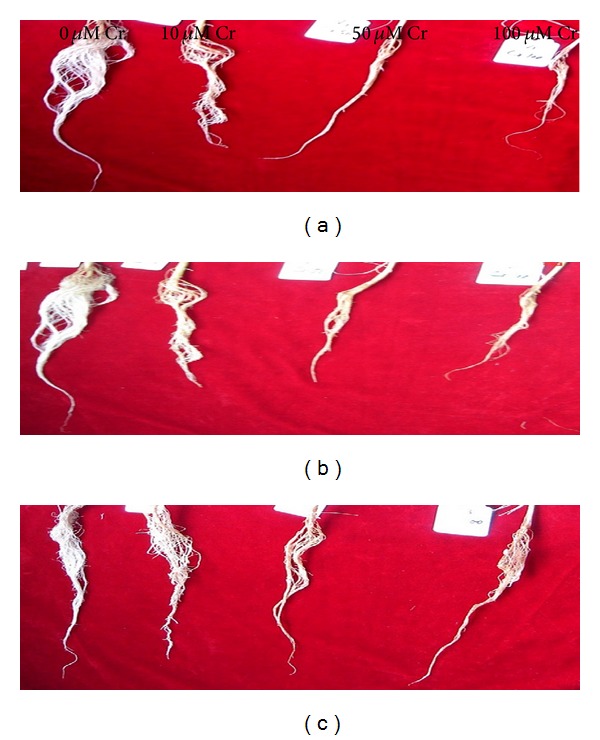
Root growth responses of herbicide resistant transgenic cotton cultivar (J208) (a), insect resistant transgenic cotton cultivar (Z905) (b), and their hybrid line (ZD14) (c) under exceeding levels of Cr.

Figure 1 shows the root morphology of both transgenic cotton cultivars (J208, Z905) and their hybrid line (ZD14) grown in Cr added nutrients substrate. Root morphology of these cultivars was influenced both in dose-dependent and genotype-dependent manners. In response to 10 *μ*M Cr, reduction in number of root hairs and secondary roots was less. However, both 50 and 100 *μ*M Cr levels demonstrated significant reduction in root lengths and number of secondary roots. J208 was the most sensitive in terms of root morphology, while ZD14 was the least affected cotton hybrid cultivar when exposed to different concentrations of Cr. Moreover, in all cultivars, there were marked reductions in number of root hairs and root vigor as well as induced root pale color at all exceeding levels of Cr compared with control. Degenerative effects regarding root morphology in all these cultivars were in the order of ZD14 < Z905 < J208.

### 3.2. Cr Stress Variably Decreased the Root Growth Performance

Measurements of growth and biomass performance show significant reduction at 5% probability level in our experimental cultivars (Table 1). Consistent decrease in the root-related mean data reveals that roots were considerably influenced in all these cultivars. As compared to related controls, root length was significantly reduced by 48% (Z905), 32% (J208), and 8% (ZD14) at exceeding levels of Cr in comparison with relevant controls. Statistically significant decline was observed at 100 *μ*M Cr, which was 71% in Z905 followed by 51% in J208 and 15% in ZD14. Compared with related controls, shoot lengths reduced with concomitant increase in Cr levels. At 5% probability level, distinctive reduction in shoot lengths was in order of Z905 (52%) > J208 (36%) > ZD14 (2.3; 34%) relative to their respective controls.

Mean data of Table 1 further reveals reduction in root biomass as well as upregulation in the mean values of biomass-based inhibition. Irrespective of the genotype, root biomasses decreased in Cr-stressed plants in dose-dependent manner. Reduction in fresh biomass reduction (collectively 184%) was more than dry biomass (collectively 165%). In comparison with their controls, marked decline was found in Z905 (89%) at 100 *μ*M Cr, while insignificant decrease was observed in J208 (37%) at 10 *μ*M Cr. Analysis of biomass-based growth inhibition exhibited increase in their mean data at higher Cr stress levels in all experimental cultivars. By comparing the fresh and dry biomass-based growth inhibition, it is evident that fresh biomass was inhibited more (59%) than dry biomass (50%). Intervarietal comparison depicts that fresh biomass-based inhibition was more in Z905 (68%) in comparison with the other two cultivars (ZD14, 59%; J208, 51%), while dry biomass-based inhibition was in the order of J208 (58%) > Z905 (57%) > ZD14 (36%) in comparison with related controls.

### 3.3. Tolerance of Cotton Cultivars Based on Their Physiological Parameters

Physiology-based tolerance studies show varied genotype-dependent responses (Table 2). Mean tabulated data depict that tolerance of both transgenic cotton cultivars and their hybrid line decrease upon exposure to Cr when compared with controls. In case of root length tolerance index, relative decrease was more in Z905 (70%) than in J208 (51%) and ZD14 (36%) at 100 *μ*M Cr. Plant height tolerance was more in ZD14 followed by J208 and Z905, while root fresh weight tolerance was more in J208 than in ZD14 and Z905, respectively.

Tabulated data further reveal that water contents-based tolerance was more in J208, while the other two cotton cultivars showed a decline, which was 5.93% in Z905 and 4.78% in ZD14. In Z905 and ZD14 the water contents decreased (data not shown), which indirectly reduced their tolerance towards exceeding levels of Cr.

### 3.4. Cr Accumulation Capacity in Roots and Its Translocation to Shoots

Table 3 shows the Cr levels in roots and shoots of both transgenic cotton cultivars and their hybrid line. According to mean tabulated data, Cr uptake in both roots and leaves was in dose-dependent manner. Statistically significant enhancement was found in all experimental cultivars with the increase in the external application of Cr. The mean data revealed that roots accumulated more Cr as compared with leaves. Greater Cr concentration was found in roots of Z905 in comparison with the other two cotton cultivars. Remarkable relative increase was found in roots of ZD14 followed by Z905 and J208, respectively. Taken together, the Cr contents in both leaves and roots, greater concentrations were found in ZD14 followed by Z905 and J208.

Table 3 furthermore shows the translocation efficiency of both parent lines and their hybrid line. In all cultivars, the translocation factor (TF) values were < 1. In J208 and ZD14, they concomitantly increased with the increase in the external application of Cr as revealed by relative increase in their mean values, while there was a decrease in TF in Z905.

### 3.5. Cr Stress-Induced Ultramorphological Changes in Roots

**Figure 2:**
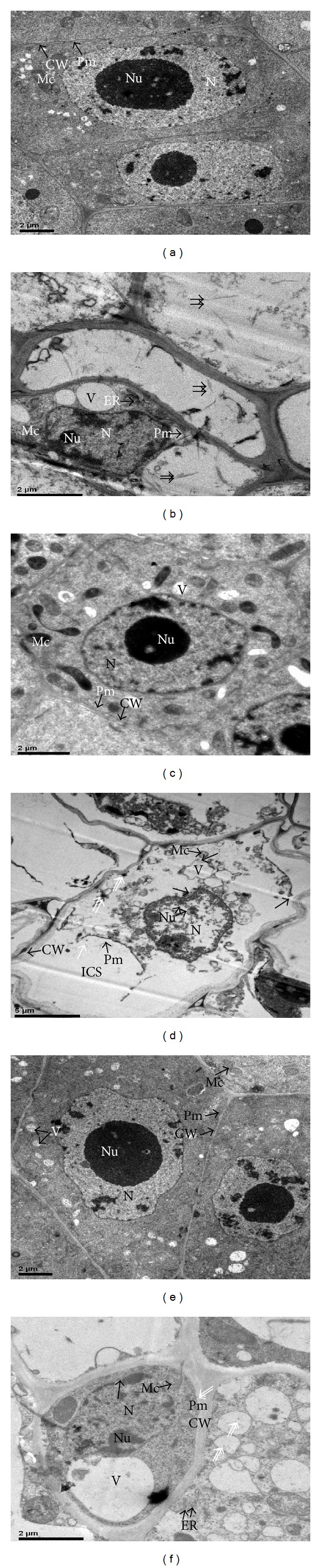
Electron micrographs of herbicide resistant transgenic cotton cultivar (J208) ((a) (CK); (b) (100 *μ*M Cr)), insect resistant transgenic cotton cultivar (Z905) ((c) (CK); (d) (100 *μ*M Cr)), and their hybrid line (ZD14) ((e) (CK); (f) (100 *μ*M Cr)) under exceeding levels of Cr. CW = cell wall; Pm = plasma membrane; Mc = mitochondria, N = nucleus; Nu = nucleoli; V = vacuole; ER = endoplasmic reticulum; ICS = intracellular spaces. Double arrows indicate Cr dense granules while single arrow shows rupturing of membranous structures.

Both genotype-dependent and concentration-dependent ultrastructural alterations were observed in root tip cells of the experimental cultivars (Figures 2(a)–2(f)). Comparative ultrastructural studies of our experimental cultivars reveal that the control cells were having typical ultrastructures (Figures 2(a), 2(c), and 2(e)). Intercellular spaces were absent. Plasma membrane was intact with the cell wall. Cell wall size was normal. There was a dense cytoplasm, with numerous mitochondria. Vacuoles were less in number and their size was normal. Nucleus was almost round-shaped having chromatin materials as well as nucleolus. However, genotype-dependent ultrastructural alterations were observed in the Cr-treated samples in all three cultivars (Figures 2(b), 2(d), and 2(f)). Less ultrastructural modifications could be observed in their hybrid line (Figure 2(f)). As a whole, the number of vacuoles increased, shape of nuclei was irregular, and cytoplasm and nucleus were less dense. Moreover, plasmolysis of the plasma membrane was evident and there was an increase in the cell wall size, disruption of plasma, and nuclear membrane as well as the number of nucleoli increased. Also, dense precipitates, most probably Cr, could be noticed in vacuoles and attached to the cell wall. Even in some micrographs, there could be found ruptured mitochondrial membranes. Such alterations were more in J208 followed by Z905 and their hybrid line (ZD14).

### 3.6. Cr Caused Oxidative Stress as Revealed by Increase in MDA and H_2_O_2_ Contents and Decrease in Total Soluble Proteins

**Figure 3:**
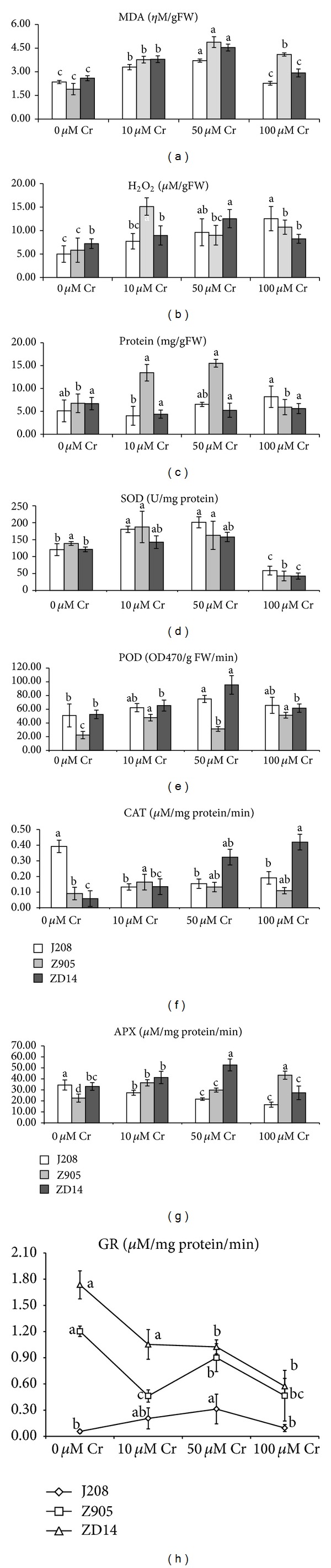
Contents of MDA (a), H_2_O_2_ (b) and total soluble proteins (c), activities of SOD (d), POD (e), CAT (f), APX (g), and GR (h) in roots of herbicide resistant transgenic cotton cultivar (J208), insect resistant transgenic cotton cultivar (Z905), and their hybrid line (ZD14) under exceeding levels of Cr.

We also investigated the biomarkers of oxidative stress such as MDA, H_2_O_2,_ and total soluble proteins (Figures 3(a)–3(c)). Mean data regarding MDA contents (Figure 3(a)) and H_2_O_2_ (Figure 3(b)) revealed that Cr-induced increments were invariably significant. For example, the MDA contents were statistically significant at 10 and 50 *μ*M Cr in comparison with related controls in both transgenic cotton cultivars and their hybrid line. However, at 100 *μ*M Cr, the MDA contents of Z905 were significantly higher than its control (i.e., 116% relative to control). Taken together, the MDA contents increased by 200% over the controls and they were found in higher amount (124%) in Z905. However, such trend could not be established in all the experimental cultivars as revealed by the mean data of H_2_O_2_ contents in roots. In J208, enhancement was regular starting from control up to the highest Cr level. At 100 *μ*M Cr level, mean data showed 151% increase over its relevant control. In Z905, significant and highest increase (160%) could be observed at 10 *μ*M over the control, which was followed by a sharp decline. However, overall an increase was recorded in H_2_O_2_ contents over its control. Like J208, similar trend was found in ZD14 up to 50 *μ*M Cr. However, at 100 *μ*M Cr level, there was a decline over the other two lower levels of Cr but its contents were higher (14%) than the controls.

Figures 3(a)–3(c) further depict that the soluble protein contents (Figure 3(c)) in roots of Cr challenged cotton cultivars. In both parent lines, a sequential increase in their mean values except at 10 *μ*M Cr in J208 and at 100 *μ*M Cr in Z905 was observed. Moreover, greater relative increase (60.18%) was found in J208 at 100 *μ*M Cr, while in Z905 such increase (129.1%) was found at 50 *μ*M Cr. In their hybrid line, decline was noticed, which was higher at 10 *μ*M Cr (i.e., 34%). However, decline was statistically nonsignificant at 5% probability levels. As a whole, percent relative increase was found higher in Z905 followed by J208 and ZD14. Furthermore, highest enhancement was noted in H_2_O_2_ (237%), while it was 200% and 70% in MDA and protein, respectively.

### 3.7. Differential Responses of ROS-Scavenging Enzymatic Antioxidants

Figures 3(d)–3(g) show the metabolic status of various enzymatic antioxidants in cotton cultivars under Cr stress. Regarding SOD activity (Figure 3(d)), the mean values showed concentration-dependent increase in all experimental cultivars up to 50 *μ*M Cr. Up to this level, gradual increase could be observed in both J208 and their hybrid line (ZD14) up to 50 *μ*M Cr, while in Z905 SOD activity first increased (35%) at 10 *μ*M Cr and then decreased but was higher than its control. At 100 *μ*M Cr, its activities significantly decreased. Highest decline was noted in Z905 (69%) as compared to its hybrid line (65%) and J208 (51%). Taken together, regarding relative increase or decrease values, the overall SOD activity was decreased by 6% in Z905 and ZD14 while, in J208, it was enhanced by 22%. However, as whole the mean data reveal 11% relative increase over the controls in SOD activity in these cultivars.

Figures 3(d)–3(g) also show considerable stimulation of the peroxidase activity (Figure 3(e)) in Cr-stressed roots over the related controls. Significant enhancement could only be observed in Z905, while the increase was almost statistically nonsignificant in the other two experimental cotton cultivars. Moreover, in J208, highest relative increase (47%) was found at 50 *μ*M Cr, while, in Z905 and ZD14, it was 128% and 82% at 100 *μ*M Cr, respectively. Overall performance of these cultivars was in the order of Z905 (33%) > ZD14 (41%) > J208 (33%).

Figures 3(d)–3(g) depict differential changes in APX activity (Figure 3(f)) upon application of Cr in the nutrient solutions. In J208, its activity gradually decreased by 37% relative to control, while, in Z905, it is significantly enhanced by 62% over its control. However, in their hybrid line, increase was significant up to 50 *μ*M Cr and at 100 *μ*M Cr there was a sharp decline by 17%. Furthermore, statistical significance was observed at all levels of Cr over the control in Z905, while in J208 a nonstatistical significance was present between 50 and 100 *μ*M Cr. In ZD14 such situation could be found between 0 and 10 as well as between 0 and 100 *μ*M Cr levels.

The spectrometric analysis of root CAT activity (Figure 3(g)) shows almost similar trend-like APX in both transgenic cotton cultivars and their hybrid line except in ZD14, which showed sharp increase at all levels of Cr over its control. Significant inhibition of CAT activity (51% relative to control) in roots of J208 could be found at 100 *μ*M Cr over the control, while significant increase (81, 614%) was found at 10 and 100 *μ*M Cr in Z905 and ZD14, respectively.

By comparing the overall performance of our present experimental cultivars, we can find that ZD14 excels the other two cultivars. Comparison of the overall activities of enzymatic antioxidants shows that there was an increase in their activities, which was in the order of CAT (387% relative to control) > POD (168% relative to control) > APX (48% relative to control) > SOD (11% relative to control).

### 3.8. Nonenzymatic Antioxidants' Response towards Cr Stress Levels

Responses of both transgenic cotton cultivars and their hybrid line with respect to nonenzymatic antioxidant such as glutathione reduced were quite different from one another (Figure 3(h)). The mean data revealed either increase as in case of J208 or decrease as in case of Z905 or both increase and decrease as in case of ZD14. GR activity in J208 first increased up to 50 *μ*M Cr (by 457%) and then decreased at 100 *μ*M Cr (by 70%). However, the value was still higher at 100 *μ*M Cr as compared with the control. In case of Z905, GR activity showed downward trend, which was highest (78%) at 10 *μ*M Cr. Regarding ZD14, GR activity at 10 *μ*M Cr increased by 11% and then gradually deceased as compared with control. Moreover, at 5% probability level, statistically significant increase could only be noticed at 50 *μ*M Cr in J208 as compared with its control, while such significance was found in Z905 at 10 and 50 *μ*M Cr as compared with control. Varietal comparative performance reveals overall an increase in GR activity by 265% over the control in J208, 65 and 49% decline in Z905 and ZD14, respectively, could be observed.

## 4. Discussion

Chromium (Cr) is a toxic element to higher vascular plants [36], which causes the oxidative damages to DNA, RNA, proteins, and pigments [8, 37]. Plants provide a unique set up of antioxidant enzymes [38] against such oxidative stress.

In the present experiment, Cr variably influenced the root morphology of cotton cultivars. Dose-dependent reduction in number of root hairs and secondary roots was found. Roots of both parent lines (J208, Z905) were poorly developed with brown coloration. Such observations have also been demonstrated by [17] in* Zea mays *L. [39], in* T. aestivum,* and [40] in* P. sativum* in Cr-stressful conditions. Pale color and stunted growth of roots might be due to interaction of Cr with various unknown root metabolic processes.

Decrease in root growth in terms of length and biomass has been a well-documented effect of Cr in plants [41]. Its presence in roots may exclude the translocation of essential metals such as Fe, S, and Zn to aerial parts of the plants thus causing indirectly the lowering down of shoot growth. A consistent decrease was found in root growth and biomass. Root fresh biomass reduced significantly as compared with root dry biomass. Similar trend was found in biomass-based growth inhibition, which exhibited an increase in their mean data at higher Cr stress levels in all experimental cultivars. Dose-dependent inhibition was noted in root-related growth parameters, which is in line with the findings of [12] in paddy rice [17], in* Z. mays* [40], in* Pisum sativum* [42], and in* B. oleracea*. Cr-induced decrease in root growth might be either due to an extension in cell cycle, which leads to arrested cell division, cell elongation, and lowering down of mitotic index [12, 43], or incapability of the roots to absorb water and nutrients from the medium [44]. Tolerance indices based on root length and fresh weight are commonly used to quantify plant metal tolerance [45]. Physiology-based tolerance indices overall declined, which was in the order of root fresh weight > root length > plant height > water contents. Mean data depict greater decrease in tolerance in root length of Z905. Further, the water contents-based tolerance was upregulated in J208, while the other two cotton cultivars showed a decline. It conveys a message that Cr caused an insignificant water stress in Z905 and ZD14. Similar results were obtained by [27] during their studies on cotton seedlings under Cd stress. However, Reisinger et al. [46] found increased tolerance in transgenic mustard cultivars.

Heavy metal uptake by roots and its translocation to aerial parts are two important indicators in phytoextraction technology. This signifies that such plant species should be selected by phytoextraction technologists, whose aerial parts are utilized and have no role in our food chain. Cotton, being mostly grown as a fiber crop in most parts of the world, is an ideal species to be used for Cr extraction and uptake. In the present experiment, Cr uptake in both transgenic cotton cultivars and their hybrid was concentration-dependent. More Cr was accumulated in roots than in leaves in all these cultivars. Taken together, the Cr uptake efficiency of both roots and leaves, Cr uptake in roots was more in Z905 as compared with other cultivars. And this could the reason that there was a greater distortion in its plasma membrane integrity as evident from electron micrographs. Similar to our findings, increase in root Cr levels has been found in rice [22],* Typha* [47], and pea [48].

Furthermore, the data about translocation factor reveal that the Cr translocation efficiency was < 1 in all these cultivar. This conveys a message that Cr was not efficiently translocated from root to shoot. That is why Cr mainly accumulated in roots than leaves. Translocation efficiency was higher in ZD14 as compared with its parent lines as revealed by relative increase in its values. Lower Cr translocation from roots to leaves is a good sign that all these cultivars can be exploited for Cr uptake purpose. Low levels of Cr translocation have also been found by [13, 49].

Typical ultrastructures were found in root tip cells of the experimental cultivars in control cells. However, at highest Cr concentration level, noticeable modifications were observed. Less ultrastructural changes were observed in hybrid line as compared to its parent lines. Increase in number of nuclei is an indicator of increased stress tolerance in respective experimental material. Such increase may result in enhanced protein synthesis [27]. Moreover, Cr dense granules were obviously present in vacuoles and attached to the cell wall. Increase in number of nuclei and vacuolar size and its number as well as the presence of Cr dense precipitates in the dead parts of the cell in all these cultivars shows a positive indication that they can be used for phytoremediation purpose in Cr-contaminated areas. Consistent with our results, Panda [22] in rice seedlings, Speranza et al. [50] in the pollen grains of kiwi, and Eleftheriou et al. [51] in* Allium cepa* observed almost similar ultramorphological features in Cr-treated root samples.

In plants, Cr may induce oxidative stress, which results in lipid peroxidation and oxidative damage [52]. Mean data regarding MDA contents revealed that they were significantly increased over the related controls in all three cultivars and were found higher in Z905. However, such trend was not found in H_2_O_2_ contents of roots. However, overall an increase was recorded in its contents over its control. Increase in MDA contents has also been reported by [53] in germinating pollen of kiwi and* M. sinensis* [54]. In contrast to our findings, there was no change in the MDA content in* S. natans* plants growing in Cr-rich wastewater [55]. An overall increase was also recorded in H_2_O_2_ contents of roots, which are in line with the findings of [22, 54, 56].

Total soluble protein contents in roots were increased in our experimental materials as a whole. Enhancement in the total soluble proteins is a good indicator that these cultivars are capable to withstand Cr-stressed environment. Another possible reason for such increase might be due to increase in the number of nuclei, which might have led an increase in the mRNA synthesis. Our results are against the findings of Ganesh et al. [57].

In our present experiment, we studied ROS-scavenging antioxidants. The SOD activity as a whole increased up to 50 *μ*M Cr, while, at 100 *μ*M Cr, its activity significantly decreased in all cultivars. Peroxidase (POD) activity in Cr-stressed roots considerably stimulated over the related controls, which was significant in Z905. The APX activity differentially changed. In J208, its activity gradually decreased, while, in Z905, it was significantly enhanced. However, in their hybrid line, increase was found up to 50 *μ*M Cr, while at 100 *μ*M Cr there was a sharp decline. CAT activity was almost similar to APX except in their hybrid line (ZD14). Our experimental cultivars showed differential responses regarding glutathione reductase activity. As a whole, activities of ROS-scavenging antioxidants increased upon exposure to Cr exceeding levels. Such increase has also been found by Liu et al. [58] in* P. sativum* under Cr stress. However, contrasting results were reported by [22] in rice and [55] in Cr hyperaccumulator* S. natans*.

Taken together, an overall increase in the activities of these enzymes reveals that SOD might have been actively involved in H_2_O_2_ production. This hypothesis can be proved by increase in H_2_O_2_ contents in roots of all these cultivars. And more importantly to detoxify H_2_O_2_, ROS-scavenging enzymes were active as a whole. This might be one of the reasons that these cultivars grew well in Cr-stressful conditions and their Cr uptake by roots was appreciable.

## 5. Conclusions

Based on our present results, it is evident that Cr influenced the physiology of our experimental cotton cultivars. However, their growth and ultramorphology were least affected. Also, there was an increase in number of nuclei and vacuoles as well as presence of Cr-dense granules in root cells. Antioxidative metabolism was also upregulated. Increase in number of nuclei and vacuoles, rise in antioxidant enzymatic activity, lower translocation factor, and higher accumulation of Cr in roots than in leaves show that these cultivars have greater potential of Cr uptake and can be considered potential candidates for reclamation of Cr-polluted areas.

## Figures and Tables

**Table 1:** Growth performance of both transgenic cotton cultivars and their hybrid line under chromium stress.

Variety		Growth performance					
	Cr levels (*μ*M)	Length (cm/plant)		Biomass (g/plant)		Growth inhibition	
		Root	Shoot	Fresh	Dry	Fresh biomass	Dry biomass
J208	0	35.83 ± 2.57^a^	47.20 ± 3.93^a^	8.32 ± 1.55^a^	0.67 ± 0.23^a^	0.00 ± 0.00^b^	0.00 ± 0.00^c^
	10	31.37 ± 1.70^b^	44.57 ± 2.29^a^	5.21 ± 1.35^b^	0.43 ± 0.22^ab^	34.61 ± 24.51^ab^	37.93 ± 20.69^b^
	50	24.00 ± 2.00^c^	34.43 ± 2.67^b^	3.85 ± 1.74^bc^	0.21 ± 0.00^b^	49.59 ± 33.52^a^	66.33 ± 10.48^a^
	100	17.60 ± 2.75^d^	30.00 ± 1.74^b^	2.34 ± 1.06^c^	0.19 ± 0.00^b^	69.41 ± 20.37^a^	69.54 ± 9.48^a^

Z905	0	22.67 ± 2.52^a^	30.20 ± 1.71^a^	7.06 ± 2.16^a^	0.50 ± 0.13^a^	0.00 ± 0.00^c^	0.00 ± 0.00^c^
	10	18.83 ± 1.76^b^	24.57 ± 2.38^b^	3.82 ± 1.47^b^	0.35 ± 0.10^ab^	45.88 ± 11.98^b^	28.71 ± 18.05^b^
	50	10.07 ± 0.90^c^	22.17 ± 3.82^b^	1.95 ± 0.77^bc^	0.19 ± 0.10^bc^	69.26 ± 16.39^a^	62.29 ± 13.42^a^
	100	6.67 ± 0.76^d^	14.60 ± 1.22^c^	0.78 ± 0.30^c^	0.10 ± 0.05^c^	87.76 ± 7.64^a^	79.57 ± 8.04^a^

ZD14	0	27.33 ± 2.52^a^	41.57 ± 3.98^a^	6.53 ± 2.10^a^	0.45 ± 0.18^a^	0.00 ± 0.00^c^	0.00 ± 0.00^b^
	10	24.87 ± 3.80^ab^	42.60 ± 2.85^a^	3.66 ± 0.44^b^	0.35 ± 0.10^ab^	38.72 ± 25.55^b^	16.26 ± 29.45^ab^
	50	20.33 ± 2.52^bc^	32.87 ± 1.21^b^	2.40 ± 0.49^bc^	0.27 ± 0.10^ab^	59.05 ± 20.08^ab^	32.88 ± 37.45^ab^
	100	17.40 ± 1.51^c^	27.50 ± 2.30^c^	1.23 ± 0.50^c^	0.17 ± 0.05^b^	80.69 ± 7.92^a^	58.25 ± 17.23^a^

Values are the means ± SD of three replications. Variants possessing the same letter are not statistically significant at *P* < 0.05.

**Table 2:** Tolerance indices of both transgenic cotton cultivars and their hybrid line under Cr stress.

Variety	Cr levels (*μ*M)	Root length	Plant height	Root fresh weight	Root water contents
J208	0	1.00 ± 0.00^a^	1.00 ± 0.00^a^	1.00 ± 0.00^a^	1.00 ± 0.00^a^
	10	0.88 ± 0.07^a^	0.91 ± 0.06^a^	0.66 ± 0.24^ab^	1.00 ± 0.00^a^
	50	0.67 ± 0.10^b^	0.70 ± 0.04^b^	0.51 ± 0.33^b^	1.02 ± 0.05^a^
	100	0.49 ± 0.06^c^	0.57 ± 0.06^c^	0.31 ± 0.20^b^	0.99 ± 0.05^a^

Z905	0	1.00 ± 0.00^a^	1.00 ± 0.00^a^	1.00 ± 0.00^a^	1.00 ± 0.00^a^
	10	0.84 ± 0.16^a^	0.82 ± 0.10^b^	0.54 ± 0.54^b^	0.98 ± 0.00^a^
	50	0.45 ± 0.05^b^	0.61 ± 0.09^c^	0.31 ± 0.31^c^	0.94 ± 0.12^a^
	100	0.30 ± 0.07^b^	0.40 ± 0.06^d^	0.12 ± 0.12^c^	0.90 ± 0.13^a^

ZD14	0	1.00 ± 0.00^a^	1.00 ± 0.00^a^	1.00 ± 0.00^a^	1.00 ± 0.00^a^
	10	0.92 ± 0.22^ab^	0.98 ± 0.09^a^	0.61 ± 0.26^b^	0.98 ± 0.07^a^
	50	0.75 ± 0.07^bc^	0.77 ± 0.02^b^	0.41 ± 0.20^bc^	0.96 ± 0.11^a^
	100	0.64 ± 0.05^c^	0.65 ± 0.02^c^	0.19 ± 0.08^c^	0.91 ± 0.03^a^

Tolerance index of the above parameters was calculated as TI = mean values in treatment/mean values in control. Mean values of TI =/> 1 show the tolerance behavior of the cultivar, while mean values of TI < 1 show the susceptibility of the cultivar towards Cr stress. Values are the means ± SD of three replications. Variants possessing the same letter are not statistically significant at *P* < 0.05.

**Table 3:** Cr concentration levels in roots and leaves and its translocation from roots to shoot in experimental cotton cultivars.

Variety	Cr levels (*μ*M)	Cr uptake (mg/gDW)		Translocation factor (TF)
		Leaf	Root	
J208	0	0.03 ± 0.01^d^	0.06 ± 0.03^d^	0.65 ± 0.40^a^
	10	0.64 ± 0.08^c^	23.07 ± 2.92^c^	0.03 ± 0.00^b^
	50	0.92 ± 0.03^b^	38.95 ± 4.45^b^	0.02 ± 0.00^b^
	100	1.08 ± 0.13^a^	59.60 ± 0.00^a^	0.02 ± 0.00^b^

Z905	0	0.05 ± 0.02^d^	1.56 ± 0.61^d^	0.03 ± 0.01^a^
	10	0.41 ± 0.07^c^	646.60 ± 29.29^c^	0.001 ± 0.00^b^
	50	1.48 ± 0.32^b^	1129.2 ± 105.98^b^	0.001 ± 0.00^b^
	100	1.97 ± 0.11^a^	1318.6 ± 150.98^a^	0.002 ± 0.00^b^

ZD14	0	0.04 ± 0.02^d^	0.09 ± 0.03^d^	0.37 ± 0.20^a^
	10	0.39 ± 0.05^c^	39.56 ± 4.06^c^	0.01 ± 0.00^b^
	50	0.52 ± 0.06^b^	55.07 ± 10.98^b^	0.01 ± 0.00^b^
	100	0.96 ± 0.00^a^	111.44 ± 7.72^a^	0.01 ± 0.00^b^

Values are the means ± SD of three replications. Variants possessing the same letter are not statistically significant at *P* < 0.05.

## Abbreviations

APX: Ascorbate peroxidase
CAT: Catalase
GR: Glutathione reductase
H_2_O_2_: Hydrogen peroxide
MDA: Malondialdehyde
POD: Guaiacol peroxidase
ROS: Reactive oxygen species
SOD: Superoxide dismutase.
